# Low-Loss and Light Substrate Integrated Waveguide Using 3D Printed Honeycomb Structure

**DOI:** 10.3390/ma12030402

**Published:** 2019-01-28

**Authors:** Yeonju Kim, Manos M. Tentzeris, Sungjoon Lim

**Affiliations:** 1 School of Electrical and Electronics Engineering, College of Engineering, Chung-Ang University, Seoul 06974, Korea; yjkim0430@cau.ac.kr; 2 School of Electrical and Computer Engineering, College of Engineering, Georgia Institute of Technology, Atlanta, GA 30332, USA

**Keywords:** 3D printing, polylactic acid, honeycomb substrate, substrate integrated waveguide

## Abstract

This article proposes a low-loss and light 3D-printed substrate-integrated waveguide (SIW). Despite the use of lossy polylactic acid (PLA) material, insertion loss is reduced, and bandwidth is increased due to a honeycomb substrate similar to air. To demonstrate the proposed concept, we fabricated microstrip-fed SIWs with solid PLA and honeycomb substrates, and compared their performance numerically and experimentally. Average measured insertion loss from 3.4 to 5.5 GHz for the honeycomb SIW is 1.38 dB, whereas SIW with solid PLA is 3.15 dB. Light weight is an additional advantage of the proposed structure.

## 1. Introduction

Device properties including low loss, low cost, light weight, and environmental compliance are essential to ensure ongoing Radio Frequency (RF) and microwave applications developments and are also important indicators to evaluate component performance in growing research areas [1,2,3,4,5].

Additive manufacturing using 3D printing [6,7,8] offers an effective alternative to implementing RF components [9,10,11] that meet the desired specifications. Fused decomposition modeling (FDM), which creates a structure by extruding a thin filament, is a representative additive manufacturing method. The main advantage of this method is that no chemical post-processing is required, which facilitates rapid prototyping and enables cost-effective and environmentally friendly production.

Common filament types for 3D printing using FDM include polylactic acid (PLA), acrylonitrile butadiene styrene (ABS), polyethylene terephthalate (PET), and polycarbonate (PC). PLA and ABS are the most popular materials [12,13,14], since both are thermoplastics and are inexpensive. ABS has average strength, flexibility, impact resistance, and heat resistance but the temperature of the print bed must be carefully controlled; whereas PLA is insensitive to the print bed temperature, and hence, is easier to print. In addition, PLA has little smell when heated and comprises biodegradable polymers. The dielectric loss of PLA is slightly larger than that of ABS [14].

The planar configuration of the substrate-integrated waveguide (SIW) makes it compatible with PCB and facilitates circuit integration with an antenna, as well as active and passive elements [15,16,17,18]. Several previous studies have considered 3D printing SIWs. For example, 3D-printed SIW cavities and interconnections manufactured using t-glase have been reported [18]. The t-glase loss tangent, tan *δ* = 0.01 at 3 GHz, is quite small compared with to PLA. However, t-glase is not biodegradable, unlike PLA. A broadband substrate integrated with a slab waveguide using polyurethane called Ninjaflex has also been proposed [19], with tan *δ* = 0.05 at 3 GHz. However, the problem of how to reduce losses caused by 3D printing materials remains.

This article proposes a honeycomb substrate design to implement a low-loss SIW, similar to air-filled SIW, using common PLA filaments for easy and environmental fabrication. Electrical properties were characterized to verify the SIW characteristics on the honeycomb substrate, and performance was compared with S-parameters for air and solid PLA filled SIW. Finally, microstrip-fed SIWs [20,21,22] with solid PLA and a honeycomb substrate are fabricated, measured, and compared.

## 2. Honeycomb Substrate Design

In this section, the electrical properties of the PLA honeycomb substrate in terms of the thickness of the structure and the frequency are verified for the purpose of designing the SIW. The hollow honeycomb geometry is well known as a structure with high mechanical strength. The minimum thickness and *T_h_* and a larger *L_h_* are required to achieve the lowest effective permittivity and lowest tangential loss of the honeycomb substrate. We set *T_h_* as 0.85 mm, which is the minimum thickness for stable 3D printing. We set *L_h_* as 2.5 mm for stable supporting of the copper tape. The ANSYS high-frequency structure simulator (HFSS, version 17.2, Pittsburgh, PA, USA) was used for electromagnetic (EM) analysis. A PLA filament provided by ColorFabb^®^ (Belfeld, The Netherlands) was used to fabricate the 3D printed substrates.

**Figure 1:**
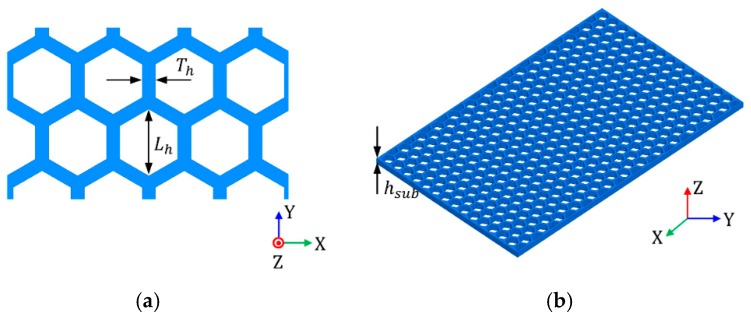
Schematic of the proposed honeycomb (**a**) unit cell, with size: *L_h_* = 2.5 mm and thickness: *T_h_* = 0.85 mm; and (**b**) substrate, with height: *h_sub_* = 1 mm.

To design the SIW using PLA material, PLA electrical properties need to be characterized for EM analysis. An infinitely large and open-ended artificial substrate can be analyzed using spectral Green’s functions [23]. However, we used the transmission line technique to characterize the effective permittivity and permeability for simplicity [24]. The dielectric constant *ε_r_* and tan *δ* of solid PLA substrate are 2.2 and 0.05 at 3.5 GHz, respectively. Based on these characteristics of the solid PLA substrate, we designed a honeycomb substrate, as shown in Figure 1.

**Figure 2:**
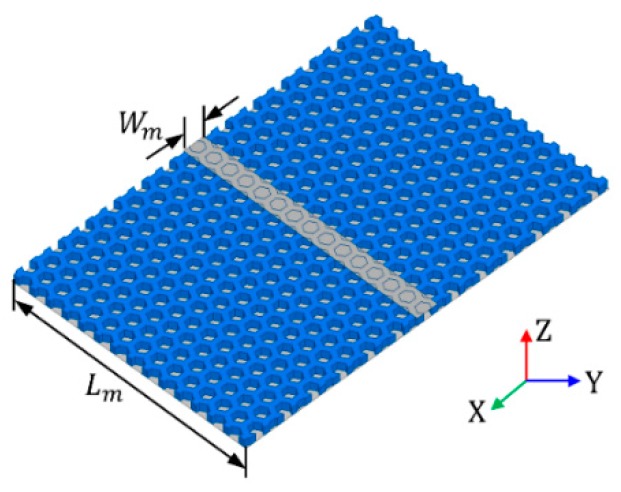
Microstrip line on the honeycomb substrate with microstrip line width, *W_m_* = 3.85 mm and honeycomb substrate length, *L_m_* = 45 mm.

A two-port simulation for microstrip line design was developed to analyze the dielectric constant of the honeycomb substrate, as shown in Figure 2. Microstrip line width *W_m_* and honeycomb substrate length *L_m_* are fixed at 3.85 mm and 45 mm for parametric study with regard to the honeycomb unit cell size *L_h_* and thickness *T_h_*, and substrate height, *h_sub_*.

**Figure 3:**
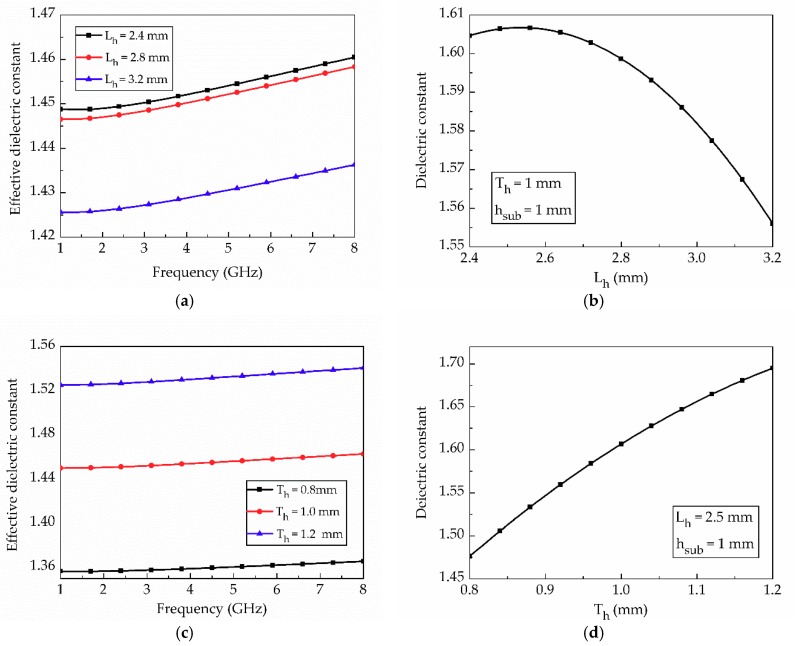

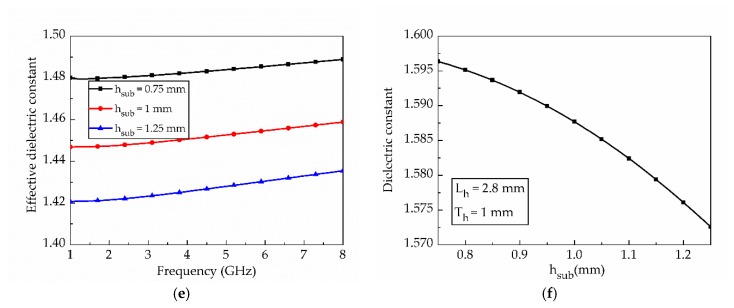
Simulated results of honeycomb substrate with microstrip line: (**a**) effective dielectric constant with respect to *L_h_*; (**b**) dielectric constant with respect to *L_h_*; (**c**) effective dielectric constant with respect to *T_h_*; (**d**) dielectric constant with respect to *T_h_*; (**e**) effective dielectric constant with respect to *h_sub_*; (**f**) dielectric constant with respect to *h_sub_*.

Substrate infill percentage depends on the honeycomb unit cell and size and determines the dielectric constant of substrate. As the size increases and the thickness decreases, the substrate dielectric constant becomes similar to that of air, as shown in Figure 3a–d. As *L_h_* increases from 2.4 to 3.2 mm, the infill percentage of honeycomb substrate decreases from 55% to 46%, and the effective dielectric constant is reduced from 1.45 to 1.43, as depicted in Figure 3a. The increase in *L_h_* also leads to a decrease in dielectric constant whose range is 1.61–1.55, as shown in Figure 3b. Figure 3c,d shows the effect of the *T_h_*. As *T_h_* increases from 0.8 to 1.2 mm, the effective dielectric constant and dielectric constant increase from 1.36 to 1.52 and from 1.47 to 1.69, respectively. According to the increase of *T_h_*, substrate infill percentage increases from 46 to 59 %, which is slightly larger than the change of infill percentage for the change in *L_h_*. Thus, the dielectric constant changes over a wider range by the change of *T_h_*. The dielectric constant is influenced by substrate height. Since the effect of the fringing field between the microstrip line and ground increases as height increases, the effective dielectric constant is decreased. As *h_sub_* increases from 0.75 to 1.25 mm, the effective dielectric constant and dielectric constant decreases from 1.48 to 1.42 and from 1.595 to 1.575, as shown in Figure 3e,f, respectively. The relation between the effective dielectric constant and dielectric constant of the microstrip line is given approximately by [25]:

(1) $$\varepsilon_{eff}=\frac{\varepsilon_{r}+1}{2}+\frac{\varepsilon_{r}-1}{2\sqrt{1+12\frac{H}{W}}}$$

where *H* is the effective height of substrate and *W* is the effective width of microstrip line.

To determine the dielectric loss for the honeycomb substrate, the T-resonator method is used [26,27]. The stub length of the T-resonator can be obtained from:

(2) $$\varepsilon_{eff,n}={\left(\frac{nc}{4L_{stub}f_{n}}\right)}^{2}$$

where *n* is the resonance index (*n* = 1, 3, 5, …), *c* is the speed of light in a vacuum, *f_n_* is the resonant frequency, and *L_stub_* is the effective physical length of the resonating stub.

**Figure 4:**
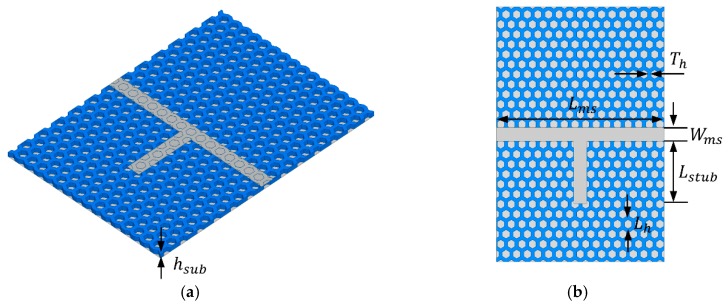
Layout of simulated T-resonator: (**a**) perspective and (**b**) top view.

**Figure 5:**
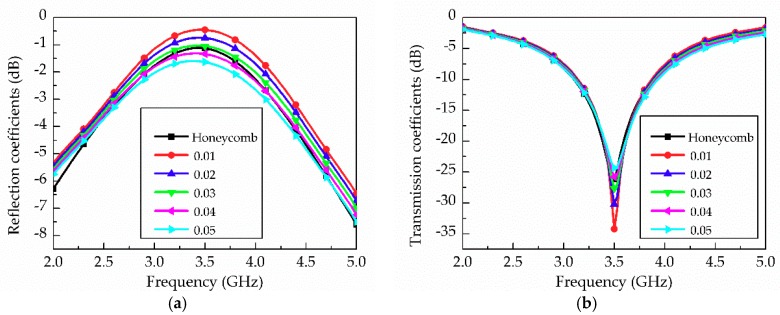
T-resonator simulation results: (**a**) reflection and (**b**) transmission coefficients when tan *δ* is varied from 0.01 to 0.05.

Figure 4 shows the T-resonator with stub *L_stub_* = 17.1 mm, microstrip feed length *L_ms_* = 70 mm, and width *W_ms_* = 3.8 mm. *L_h_* = 2.5 mm, *T_h_* = 1 mm and *h_sub_* = 1 mm are used for T-resonator design on a honeycomb substrate. In EM simulations, the effective dielectric constant and tan *δ* of the honeycomb substrate are characterized. Figure 5a,b shows the transmission and reflection coefficients of the T-resonator on the substrate whose tan *δ* varies from 0.01 to 0.05, respectively. Thus, the effective dielectric constant and tan *δ* for the specified honeycomb substrate are determined to 1.6 and 0.035 at 3.5 GHz, respectively. 

## 3. Results and Discussion

### 3.1. Honeycomb SIW Design

**Figure 6:**
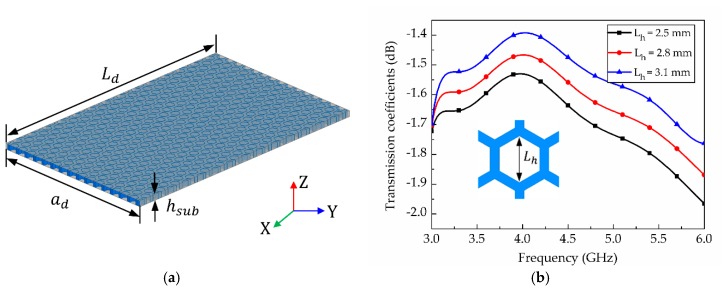

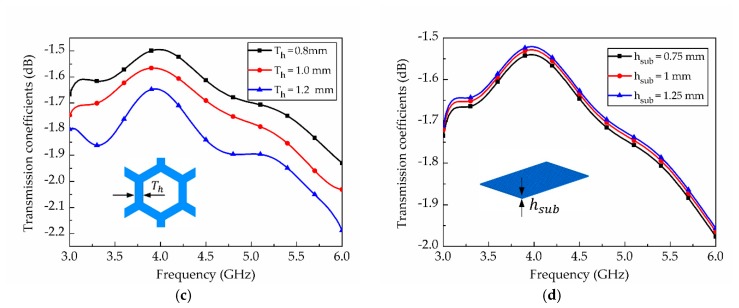
Simulated results of (**a**) SIW on honeycomb substrate; and transmission coefficients with respect to (**b**) *L_h_*; (**c**) *T_h_*; (**d**) *h_sub_*.

Parametric studies regarding *L_h_*, *T_h_*, *h_sub_* (see Figure 1) were performed to investigate insertion loss for SIW on the honeycomb substrate. Figure 6a shows the SIW geometry, with the SIW width *a_d_* = 47.3 mm and length *L_d_* = 75 mm. Figure 6b shows the insertion losses for the SIW on honeycomb substrate regrading *L_h_* and when *L_h_* was 2.5, 2.8, and 3.1 mm. It is observed that the average insertion losses of the SIW were 1.69, 1.62, and 1.54 dB when *L_h_* was 2.5, 2.8, and 3.1 mm, respectively. Figure 6c shows the insertion losses of the SIW with respect to *T_h_*. When *T_h_* was 0.8, 1.0, and 1.2 mm, the average insertion losses of the SIW were 1.66, 1.74 and 1.86 dB, respectively. Since the honeycomb unit cell’s larger *L_h_* and thinner *T_h_* (see Figure 1) encompasses more empty space, larger *L_h_* and smaller *T_h_* were preferred for lower insertion loss. When *h_sub_* was 0.75, 1.0, and 1.25 mm, the average insertion losses of the SIW were 1.7, 1.69, and 1.68 dB, as shown in Figure 6d. Substrate height, *h_sub_*, did not significantly affect SIW insertion loss compared to size *L_h_* and thickness *T_h_*. 

We used the Fused Deposition Modeling (FDM) Ultimaker 2 plus (Geldermalsen, The Netherlands) 3D printer to print the honeycomb substrate. The diameter of the 3D printer filament extrusion nozzle is 0.8 mm, and the layer resolution for the quick draft is 0.6 mm. Taking into consideration the printing limitations and the advantage of stable fabrication with the 3D printer Ultimaker 2, including the results for *L_h_* and *T_h_* for the insertion loss, *L_h_* = 2.5 mm and *T_h_* = 0.85 mm were used. Substrate height, *h_sub_*, was also considered to specify the honeycomb substrate dimension, since *h_sub_* was the effect on determining the characteristic impedance of the feeding line. In addition, a thinner SIW is preferred for the planar configuration. Therefore, *h_sub_* was set to 0.97 mm after considering the printing resolution. The final dimension of the honeycomb substrate provides an effective dielectric constant = 1.47 and tan *δ* = 0.03 at 3.5 GHz. Therefore, both dielectric constant and tangential loss were reduced compared to the PLA-filled substrate.

**Figure 7:**
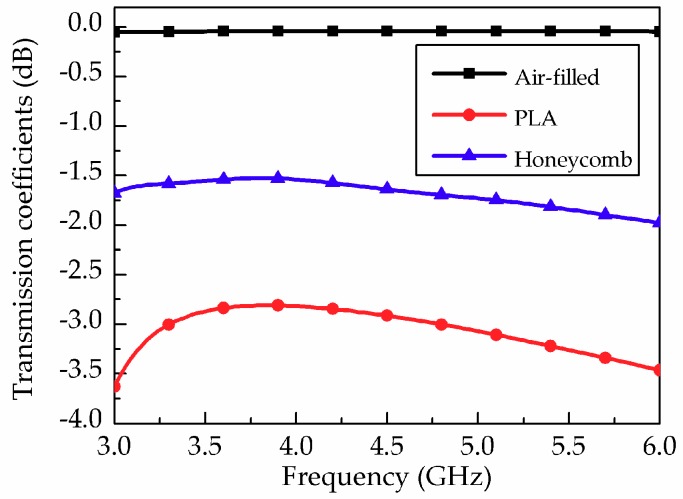
Simulated transmission coefficients of SIW with air-filled, solid PLA, and honeycomb substrates.

Based on the honeycomb substrate in Figure 1, we designed the SIW with a cut-off frequency of 2.53 GHz. To verify the insertion loss of the proposed SIW with the honeycomb, the transmission coefficient was simulated and compared with that of the SIW filled with air (empty) and solid PLA. Figure 7 shows that the average insertion losses from 3.4 GHz to 5.5 GHz were 0.04 dB, 2.96 dB, and 1.64 dB for air-filled, solid PLA, and honeycomb SIW, respectively. Table 1 compares the results of several simulated SIWs. The results demonstrate that the insertion loss can be reduced with a honeycomb structure. The insertion loss can be further reduced by minimizing the PLA frame thickness.

**Figure 8:**
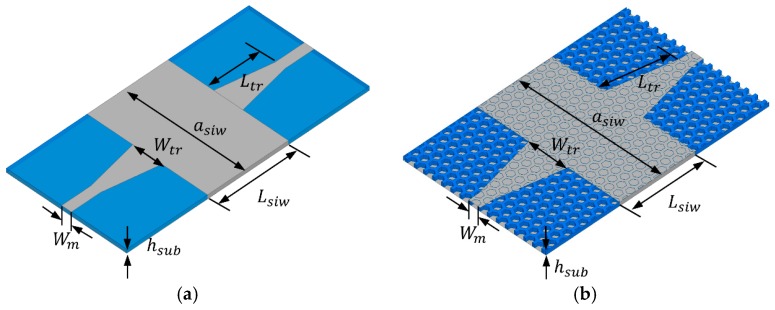
Geometry of microstrip-fed SIW with (in mm): (**a**) solid PLA substrate: *a_siw_* = 37.2, *W_m_* = 3, *L_siw_* = 25, *W_tr_* = 9, *L_tr_* = 16, *h_sub_* = 0.97; (**b**) honeycomb substrate: *a_siw_* = 47.3, *W_m_* = 3.92, *L_siw_* = 25, *W_tr_* = 12.92, *L_tr_* = 18, *h_sub_* = 0.97.

**Figure 9:**
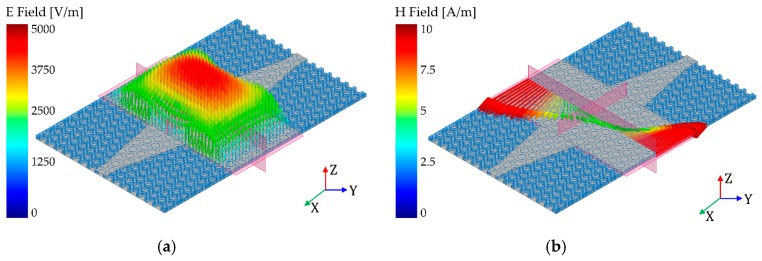

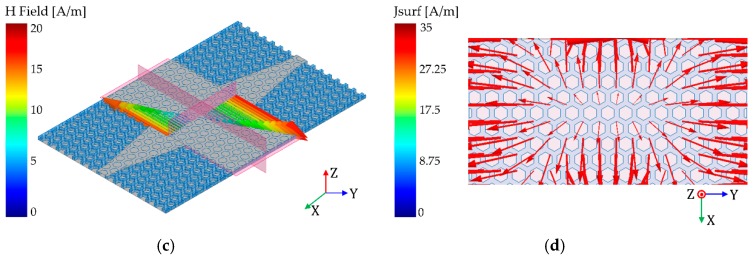
Simulated $TE_{10}$ mode SIW field distributions: (**a**) $E_{z}$ on x-y plane; (**b**) $H_{x}$ on y-z plane; (**c**) $H_{y}$ on z-x plane; and (**d**) electrical current on x-y plane at 4 GHz.

To measure the SIW, a microstrip-fed SIW was designed with a tapered transition, as shown in Figure 8. We designed a $TE_{10}$ mode SIW that has $E_{z}$, $H_{x}$, $H_{y}$ field components. Since surface currents in transverse magnetic mode (TM) are interrupted by the via, only transverse electric mode ($TE_{m0}$) can be supported in the SIW. Figure 9a–d shows the electric field distribution ($E_{z}$), magnetic field distribution ($H_{x}$, $H_{y}$) and electric current distribution on the SIW, respectively. In addition, Figure 9d shows the electric current distribution on the SIW. The electric currents are uniformly distributed on the surface of the SIW conductor, and they are at their maximum at the side because of the shorted via. Since these field distributions of SIW are similar to the microstrip line, the fields can be matched, and the device reflection response is improved. Simulation results are discussed and compared with the measurement results in the following section.

### 3.2. Microstrip-Fed SIW Fabrication and Measurement

**Figure 10:**
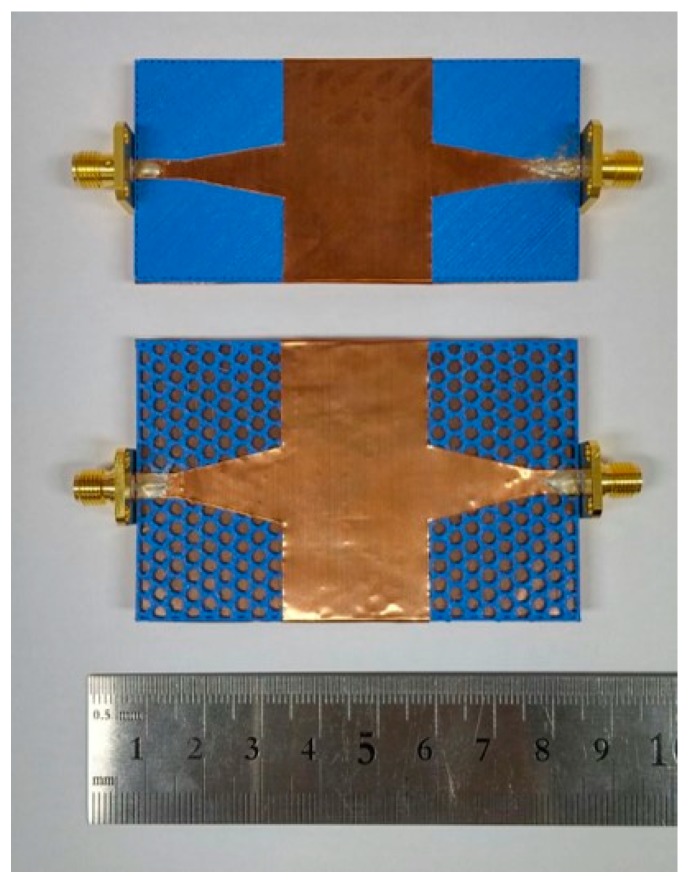
Fabricated 3D-printed microstrip-fed SIW with solid PLA and honeycomb substrate.

To demonstrate the proposed SIW performance, we fabricated two samples of the microstrip-fed SIW with solid PLA and honeycomb substrate, as shown in Figure 10. The overall substrate length and SIW length of the two samples were the same, at 75 mm and 25 mm, respectively. To have the same cutoff frequency of transverse electric *TE*_10_ mode at 2.53 GHz, the SIW width of the two samples must be different, because the effective dielectric constants of the two substrates are different. Therefore, the SIW widths of the solid PLA and honeycomb substrates were 37.2 mm and 47.3 mm, respectively. It took 30 min to 3D-print the overall structure.

Figure 8 shows the geometry of the microstrip-fed SIW with solid PLA and honeycomb substrates. The honeycomb geometry was designed in consideration of the minimum 3D-printing resolution, which is 0.1 mm. Conductive patterns are realized using copper tape, and Sub-Miniature version A (SMA) connectors are mounted using silver epoxy.

**Figure 11:**
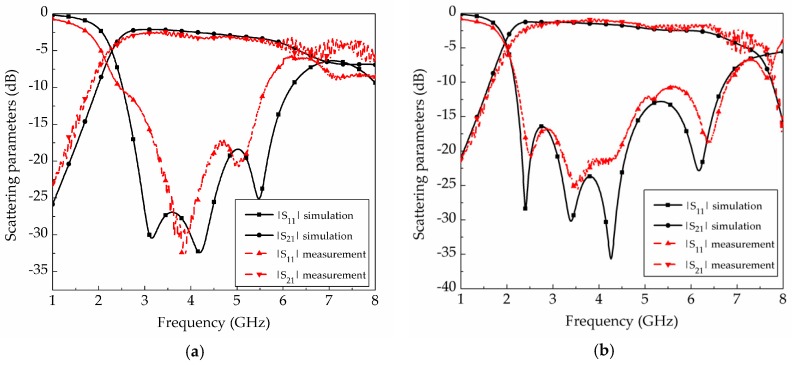
Simulated and measured results of SIWs: (**a**) solid PLA; (**b**) honeycomb.

The simulation and measurement results for the two prototypes are shown in Figure 11. The measured average insertion loss with the fabricated honeycomb substrate is 1.38 dB from 3.4–5.5 GHz, while that with the fabricated solid PLA is 3.15 dB for the same frequency range. The simulated and measured insertion losses of SIW fabricated on the solid PLA substrate are 2.7 dB and 3.15 dB within the frequency range from 3.4–5.5 GHz, respectively; whereas those of the SIW fabricated on the honeycomb substrate are 1.81 dB and 1.38 dB from 3.4–5.5 GHz, respectively. The simulated and measured 10-dB bandwidth of the SIW fabricated on the solid PLA substrate are 4.65 GHz and 3.14 GHz, respectively; whereas those of the SIW fabricated on the honeycomb substrate are 4.56 GHz and 4.57 GHz. The simulation and measurement results show good agreement despite fabrication tolerance. Table 2 shows a performance comparison, in which the weight of the SIW with the honeycomb substrate is 1.72 g, while that of SIW with the solid PLA is 3.0 g. Therefore, the insertion loss and weight of the proposed SIW with the honeycomb substrate are reduced by 56% and 43%, respectively. In addition, the 10-dB impedance bandwidth is increased from 70% to 102% compared to the SIW with the solid PLA material.

We proposed a 3D-printed SIW with honeycomb geometry which shows low insertion loss, although cheap plastic material is used. The 3D-printed SIW has low cost, light weight, and low loss compared to the PCB-based SIW. SIWs have been applied in antennas [28,29,30], circuits [31,32,33,34,35], and sensors [36,37,38]. The proposed work could be also used to various RF applications.

## 4. Conclusions

A low-loss and lightweight SIW is proposed using a 3D-printed honeycomb substrate. The proposed microstrip-fed SIW is compared to microstrip-fed SIW with solid PLA. The insertion loss of the SIW with a honeycomb substrate is reduced from 3.15 dB to 1.38 dB and the weight is reduced from 3 g to 1.7 g. Additionally, a wider fractional bandwidth (FBW) of 102% is achieved with the proposed structure. In addition, the advantages mentioned above of the proposed honeycomb SIW will be useful for space applications requiring light weight. However, the minimum resolution of 3D printing technology is higher than the conventional lithography fabrication process, and the post-processing for the conductive pattern is required in this work. In addition, the maximum frequency is limited because the dielectric loss is higher in high frequencies. Nevertheless, it is acceptable in the sub-6 GHz spectrum. With the advance of 3D printing technology, if a high-performance 3D printer is used, the resolution can be minimized, and metallic patterns can also be 3D printed. In addition, if low-loss filaments are developed in the future, the operation frequency can be increased.

## Figures and Tables

**Table 1:** Comparison of SIW characteristics between air-filled, solid PLA and the proposed honeycomb.

Characteristics	Air-Filled	Solid PLA	Honeycomb
Cutoff frequency (GHz)	2.53		
Dimension (*a_d_*) (mm)	56.8	40	47.3
Dielectric constant (*ε_r_*)	1.09	2.2	1.57
Loss tangent (tan *δ*)	0	0.05	0.03
Average insertion loss (dB)	0.04	2.96	1.64

**Table 2:** Performance comparison of SIWs with solid PLA and the proposed honeycomb.

Parameters	Solid PLA	Honeycomb
Average insertion loss (dB)	3.15	1.38
Weight (g)	3.0	1.7
FBW (%)	70	102
